# Shorter antitubercular therapy for extrapulmonary tuberculosis - a case report

**DOI:** 10.1186/s12879-023-08941-2

**Published:** 2024-01-15

**Authors:** Diviya Bharathi Ravikumar, Barath Prashanth Sivasubramanian, Ankur Singla, Rakshaya Venu, Saketh Palasamudram Shekar

**Affiliations:** 1 Internal Medicine University: ESIC MC and PGIMSR, Chennai, Tamilnadu India; 2 Infectious Diseases, University of Texas Health, San Antonio, TX 78229 USA; 3 Internal Medicine, Dayanand Medical College & Hospital, Ludhiana, Punjab 141001 India; 4 Internal Medicine, Saveetha Medical College and Hospital, Chennai, Tamilnadu India; 5 Interventional Pulmonology Pulmonary and Sleep Associates of Huntsville, Huntsville, Alabama USA

**Keywords:** Tamponade, Tuberculosis, Common variable immunodeficiency disease, Pericardial effusion, Case report

## Abstract

**Introduction:**

Extrapulmonary tuberculosis (EPTB) adds to India’s significant economic burden, with pericardial effusion being a potentially fatal complication. This case report highlights the need for early diagnosis and the feasibility of shorter-duration treatment for EPTB in developing countries.

**Presentation:**

This case report describes a 19-year-old male from Southeast Asia who had a history of bronchiectasis involving the left lower lobe and the right middle lobe, which was cystic in nature, as well as multiple episodes of non-tuberculous pneumonia. Currently, he presented with fever, hypotension, tachycardia, and acute kidney injury. Echocardiogram showed left ventricular dysfunction with a left ventricular ejection fraction (LVEF) of 45% and moderate pericardial effusion. Early signs of cardiac tamponade were noted, specifically the absence of respiratory variation in the right ventricle and left ventricle collapse. Emergent pericardiocentesis was performed, and hemorrhagic pericardial fluid was aspirated. Fluid analysis revealed high levels of LDH (5000 U/L), polymorphonuclear leukocytosis, and acid-fast bacilli that were visualized on microscopy, which led to the diagnosis of pericardial tuberculosis. A CT of the abdomen showed hepatosplenomegaly and polyserositis. Empirically, antitubercular therapy consisting of isoniazid, rifampin, pyrazinamide, and ethambutol was administered for 2 months and isoniazid along with rifampicin was given for the next 4 months. Serial echocardiograms in the following months showed an improvement in LVEF (55%) and decreased effusion. However, during this treatment period, due to frequent episodes of pneumonia, the evaluation of immunodeficiency disorders was performed and revealed low levels of IgG (4.741 g/L), IgA (0.238 g/L), and IgM (0.098 g/L). He was diagnosed with common variable immunodeficiency disease and received intravenous immunoglobulin therapy.

**Conclusion:**

This report emphasizes the timely identification of cardiac tamponade and the effective management of EPTB through a shorter-than-recommended course of antitubercular therapy, resulting in the alleviation of symptoms and better overall health outcomes.

**Supplementary Information:**

The online version contains supplementary material available at 10.1186/s12879-023-08941-2.

## Introduction

Extrapulmonary tuberculosis (EPTB) is caused by Mycobacterium tuberculosis, and can affect various organs, such as the lymph nodes, pericardium, bones, joints, central nervous system, genitourinary system, and digestive system. According to the WHO Global Tuberculosis Report 2022, in 2021, 6.4 million people had a new episode of tuberculosis. Of these, 83% had pulmonary tuberculosis, and 17% had extrapulmonary tuberculosis. Most notifications came from the African, South-East Asia, and Western Pacific regions, with the South-East Asia region contributing nearly half of all notifications [1]. In India, where extrapulmonary tuberculosis accounts for around one-fifth of all tuberculosis incidences, lymph nodes are the most common site of involvement. The prevalence of extrapulmonary tuberculosis is noteworthy and constitutes 15–20% of all tuberculosis cases in HIV-negative patients and 40–50% of new tuberculosis cases in HIV-positive individuals [2]. Although pericardial involvement is more prevalent than myocardial involvement, cardiac tuberculosis is extremely rare, affecting approximately 1–2% of tuberculosis patients [3, 4]. This can lead to medical emergencies such as pericardial effusion and cardiac tamponade, which require immediate diagnosis and treatment. In developing countries, tuberculosis is the most common cause of pericardial effusion, which can be fatal if left untreated, with a median survival time of 3.7 months [5–7]. In accordance with the WHO global tuberculosis report 2022, for individuals with drug-susceptible tuberculosis (both pulmonary and extrapulmonary), the most recent WHO World Health Organization guidelines, published in 2022, strongly advise a 6-month regimen of isoniazid (H), rifampicin (r), ethambutol (e), and pyrazinamide (Z): all four drugs for the first two months, followed by H and r for the remaining four months [1]. With proper treatment, the mortality rate can be reduced to less than 20% in immunocompetent individuals and up to 30% in patients with HIV, whereas without treatment, the mortality rate exceeds 90% [8].

Common variable immunodeficiency disease (CVID) is a primary humoral immunodeficiency illness that is characterized by decreased blood levels of immunoglobulin G (IgG), immunoglobulin A (IgA), or immunoglobulin M (IgM), recurrent sinopulmonary infections, autoimmune disorders, granulomatous diseases, an increased risk of malignancy, and an impaired antibody response despite an acceptable number of B cells due to impaired B cell differentiation [9–11]. The prevalence of common variable immunodeficiency disease, a rare disorder, has been reported to vary depending on the population studied. In Europe, the estimated prevalence is approximately 1:25,000, while in the United States, it is reported to be 1:50,000, as per recent studies [12, 13].

In this report, we describe a young adult from Southeast Asia who had established bronchiectasis in the past and is now presenting with features of cardiac tamponade due to tuberculosis. We present a case report in which new guidelines suggesting 6 months of anti-tuberculosis therapy have proven effective even in an immunocompromised individual.

## Case presentation

**Fig. 1:**
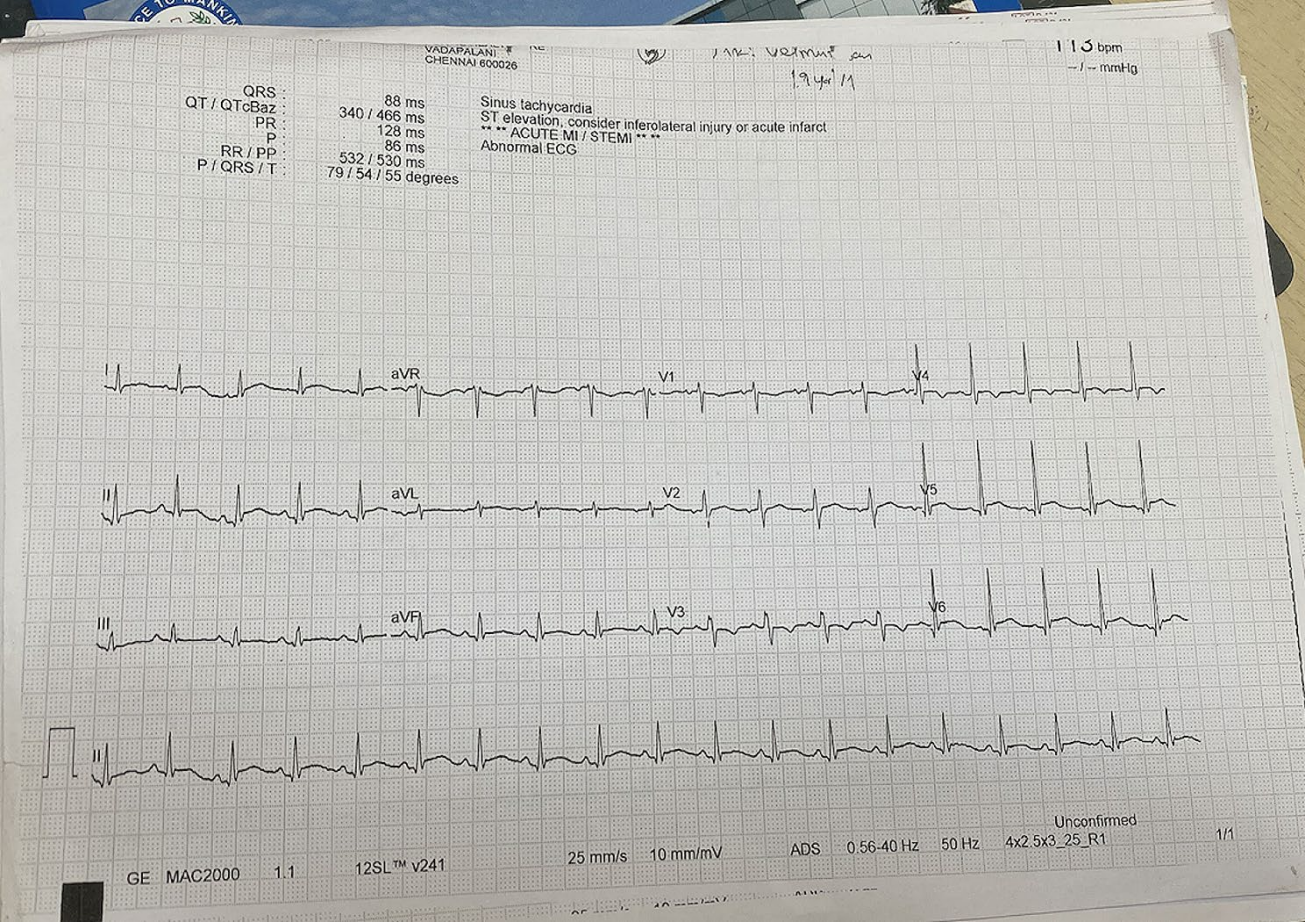
Electrocardiogram showing tachycardia, sinus rhythm, non-specific ST and T changes in leads V2 through V6

A 19-year-old male from a developing Southeast Asian nation with a history of bronchiectasis involving the left lower lobe and the right middle lobe, which was cystic in nature. Additionally, he experienced multiple episodes of non-tuberculous pneumonia in the past. Currently, he presented with hypotension, fever, cough, and vomiting for 4 days. Upon examination, the patient was conscious and oriented with no signs of pedal edema. Vital signs showed a temperature of 98.4 °F, blood pressure of 70/40 mm Hg, oxygen saturation of 96% on room air, pulse rate of 96/min, and respiratory rate of 24/min. Systemic examination was normal with no murmurs and clear bilateral respiratory sounds. An RT PCR for Covid 19 and a CT scan of the chest ruled out COVID-19 infection. An EKG showing tachycardia, sinus rhythm, non-specific ST, and T changes in leads V2 through V6 [Figure-1].

Blood investigations revealed hemoglobin of 8 gm/dl, ferritin of 362.8 µg/liter, and total iron binding capacity of 242.8 µg/dL, suggestive of anemia of chronic disease. White blood cell counts (16,200 cells/cumm) were on the higher end with a neutrophilic predominance and elevated ESR (100 mm/hr) indicative of ongoing inflammation. Blood urea nitrogen (31 mg/dl) and creatinine (4.3 mg/dl) showed a rising trend with a high urine protein creatinine ratio (UPCR) of 0.32, indicative of acute kidney injury. Alanine aminotransferase (473 U/L), Aspartate aminotransferase (185 U/L) were found to be high.

He was started on fluid resuscitation with 1 L of normal saline and inotropic support with nor-adrenaline injection. With an elevated troponin-T level (22.37 ng/mL), an echocardiogram was ordered, which showed global hypokinesia with a left ventricular ejection fraction (LVEF) of 45%, mild LV dysfunction, and moderate pericardial effusion. Early signs of cardiac tamponade, specifically the absence of respiratory variation of the right ventricle and left ventricle collapse were visualized. Due to this, an emergent pericardiocentesis was performed, and 550 ml of hemorrhagic pericardial fluid was aspirated through a subxiphoid approach. The procedure was performed again two days later, and 70–80 ml of fluid was obtained. The pericardial fluid study revealed an exudative fluid with a polymorphic predominance. The lactate dehydrogenase level (5000 IU/L) was found to be high, while the adenosine deaminase level (3 U/L) was found to be below the diagnostic range for tuberculosis (TB). MTB Xpert detected medium MTB, and Rif Resistance was not detected. Acid-fast organisms were visualized on the Ziehl-Neelsen stain. Blood and pericardial fluid cultures employing modified Middlebrook 7H9 broth as the culture media showed growth of acid-fast bacilli after 3 weeks. Table-1 shows the results of the pericardial fluid analysis.

**Table 1:** Pericardial fluid analysis

Volume	60	mL
Appearance	Turbid	
Proteins	3.6 (exudative)	gm/dL
Lactate dehydrogenase (LDH) levels	5000	U/L
Adenosine deaminase (ADA) levels	3.00	U/L
Epithelial cells	Not detected	
Polymorphonuclear leukocytes	5–7	
Ziehl-Neelsen stain	Acid fast bacilli visualized	

CT scan and ultrasound of the abdomen showed hepatosplenomegaly, diffuse gall bladder wall edema, subcapsular hematoma along the liver surface, mild ascites with 500 ml fluid, moderate bilateral pleural effusion, and bilateral hypodense kidneys indicative of acute kidney injury. The patient was diagnosed with disseminated tuberculosis. The visualization of acid-fast organisms, high LDH levels, and the presence of polyserositis led to suspicion of tuberculosis as the underlying cause. The patient was started on empirical antitubercular therapy consisting of isoniazid, rifampin, pyrazinamide, and ethambutol for 2 months, and 4 months of isoniazid and rifampicin. To prevent isoniazid-induced peripheral neuropathy vitamin B6 supplements were also administered. He demonstrated progress with consistent hemodynamics and his symptoms resolved. A serial echocardiogram performed after 3 months and 6 months showed adequate LV function with EF of 55%, and reduced pericardial effusion. Due to the improvement of cardiac function the frequency of scans was reduced. During these 6 months, the patient developed 2 more episodes of pneumonia. Considering the patient’s history of repeated hospital admissions, he was investigated for immunodeficiencies. The immunoassays showed low levels of IgG (4.741 g/L), IgA (0.238 g/L), and IgM (0.098 g/L). Targeted gene sequencing, including selective capture and sequencing of protein-coding regions, revealed heterozygous positivity for Cystic Fibrosis Transmembrane Conductance Regulator (CFTR) and Interferon-alpha/beta receptor subunit 2 (IFNAR2) genes, located on exons 17 and 9 respectively, both autosomal recessive in inheritance. However, the test results for cystic fibrosis were of uncertain significance. The results of flow cytometry depicted 66.6% CD3 + cells, 30.3% CD19 + cells, and 2.6% CD16 + CD56 + cells. The CD4 + and CD8 + cell count is 25.5% and 36.06% respectively, with a CD4/CD8 ratio of 0.71.

**Fig. 2:**
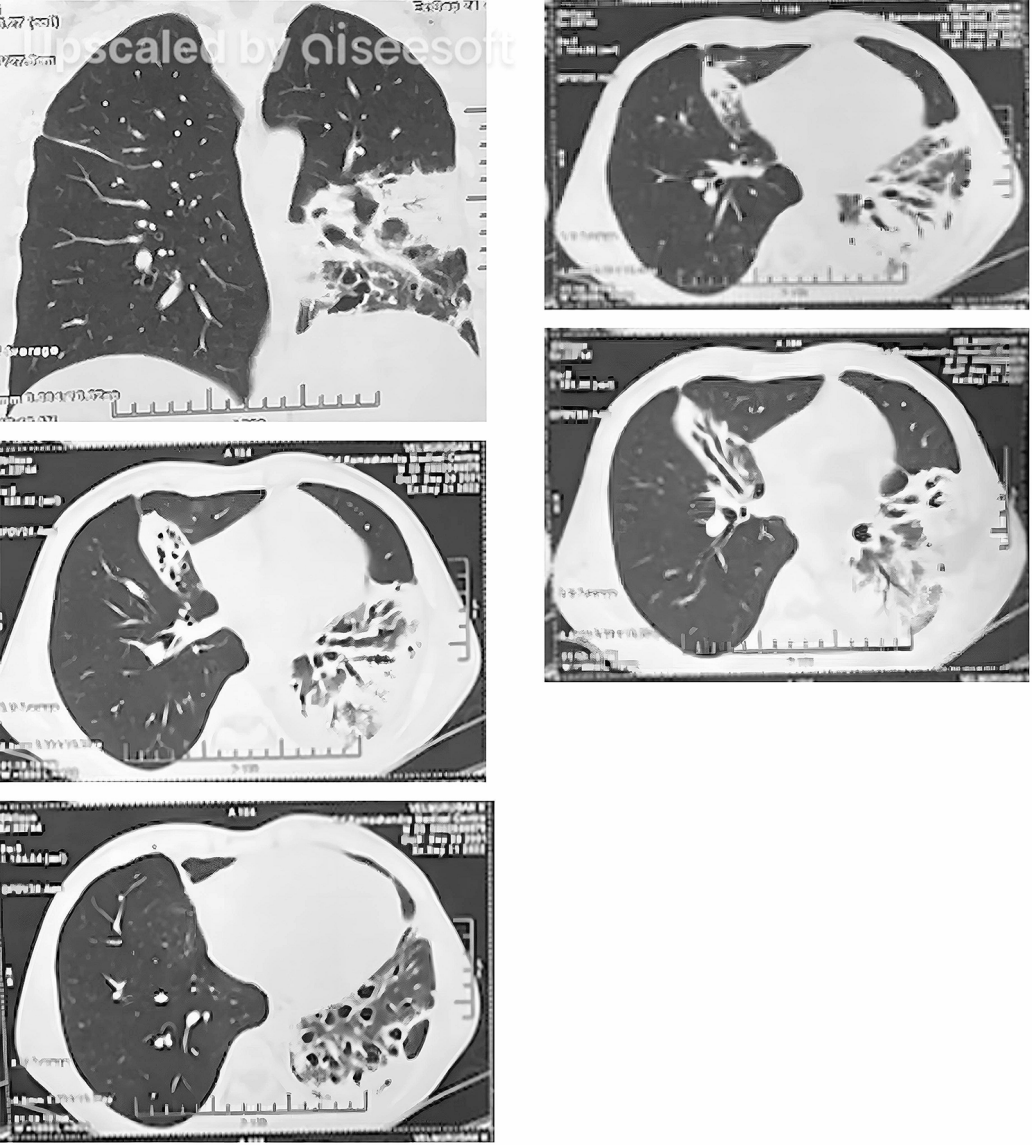
a to e: CT scan of the chest revealed varicose bronchiectasis with surrounding fibrosis and architectural distortion in the lateral segment of the right middle lobe and left lower lobe; patchy areas of consolidation with surrounding ground glass opacities in the lateral segment of the right middle lobe, posterior segment of left upper lobe and left lower lobe; few sub centrilobular nodules in the posteromedial segment of right lower lobe; mediastinal lymphadenopathy

The patient was non-reactive for HIV-1 and HIV-2 antibodies, hepatitis surface antigen for hepatitis B, and hepatitis C antibodies for hepatitis C. A CT scan of the chest showed bronchiectasis with surrounding ground glass opacities in the right middle, left upper, and left lower lobe. Multiple sub centric nodules in the right lower lobe and mediastinal lymphadenopathy were seen. Features of the CT scan were indicative of infective etiology. Bronchial washings and transbronchial biopsy revealed normal colonizers of the respiratory tract and no granulomas respectively Figure-2 shows the CT scan.

After excluding other possible etiologies, he was diagnosed with common variable immunodeficiency (CVID) and was started on intravenous immunoglobulin therapy every three weeks. Along with that, his bronchiectasis was managed with bronchodilators and chest physiotherapy. At the 24 months follow up visit, the patient was asymptomatic.

## Discussion

Cardiac tamponade results from the rapid filling of fluid within the pericardium, leading to compression of the chambers of the heart and resulting in decreased venous return, ventricular filling, and cardiac output [14]. The most significant echocardiographic findings for cardiac tamponade include the presence of a pericardial effusion, dilated IVC, and hepatic veins, indicating elevated systemic venous pressures, and a left ventricle with reduced end-diastolic and end-systolic dimensions [15, 16]. In the presentation described above, an echocardiogram shows global hypokinesia with a left ventricular ejection fraction (LVEF) of 45%, moderate pericardial effusion, and absent respiratory variation without any LV collapse which are indicative of early tamponade. Cardiac tamponade requires emergent pericardiocentesis to relieve the pressures. The diagnosis of pericardial tuberculosis can be challenging due to difficulties in obtaining adequate diagnostic samples. The visualization of the acid-fast mycobacteria from the pericardial fluid aided in identifying tuberculosis as the cause. The biochemical indicators of tuberculosis infection, particularly serum LDH levels, have a positive correlation with mycobacterial load and they can be employed in resource-limited areas [17, 18]. Similarly, in our patient, the LDH level (5000 IU/L) is found to be high in the pericardial fluid. However, LDH elevation is also noticed in lung inflammatory conditions and vasculitis. Based on low peripheral eosinophil count, and negative antinuclear antibodies and double stranded DNA, autoimmune conditions could be excluded [19]. ADA activity measurement is a commonly used diagnostic biomarker for EPTB due to the stimulation of T-cell lymphocytes by mycobacterial antigens [20]. However, in our case report, the ADA levels are below the diagnostic threshold.

A recent study from the American Heart Association observes that the majority of the data on the treatment of tuberculous pericarditis involves a four-drug regimen of antituberculosis chemotherapy, which consists of isoniazid (300 mg/day), rifampicin (600 mg/day), ethambutol (15–25 mg/kg/day), and pyrazinamide (15–30 mg/kg/day), along with corticosteroids and, in some cases, open or percutaneous drainage. The initial administration of this regimen should be continued for two months, and the same regimen is used to treat pulmonary tuberculosis. Rifampicin and pyrazinamide should be continued for another six months, irrespective of the patient’s immunological condition [20]. Reuter et al. showed that closed pericardiocentesis along with six months of antitubercular chemotherapy was found to be an effective treatment for tuberculous effusions [21]. This provides evidence that a shorter treatment plan is effective in EPTB.

## Conclusion

In conclusion, pericardial effusion is an uncommon extra-pulmonary manifestation of tuberculosis, and tamponade is even rarer. Despite its rarity, timely intervention and treatment are crucial in managing this condition. A higher degree of clinical suspicion is needed to diagnose pericardial effusion in tuberculosis patients. Moreover, a shorter duration of antitubercular therapy can be effective, even in the presence of immunocompromising conditions, such as Common Variable Immune Deficiency.

### Electronic supplementary material

Below is the link to the electronic supplementary material.

Supplementary Material 1

## Data Availability

Data generated/ analyzed in this study is included in this published article.

## References

[1] Bagcchi S (2023). WHO’s Global Tuberculosis Report 2022. Lancet Microbe.

[2] Sharma SK, Ryan H, Khaparde S (2017). Index-TB guidelines: guidelines on extrapulmonary Tuberculosis for India. Indian J Med Res.

[3] Jorquera-Román M, Araya-Cancino J, Enríquez-Montenegro J (2021). [Tuberculous pericarditis an infrequent extrapulmonary manifestation of TB]. Rev Med Chil.

[4] Johari MI, Ramli AW, Mat Lawi F, Bin Fouzi MAH, Suardi KPS (2019). A rare case of Purulent Pericardial TB. Cureus.

[5] Mayosi BM, Burgess LJ, Doubell AF (2005). Tuberculous pericarditis. Circulation.

[6] Vakamudi S, Ho N, Cremer PC (2017). Pericardial effusions: causes, diagnosis, and management. Prog Cardiovasc Dis.

[7] Imazio M, Gaido L, Battaglia A, Gaita F (2017). Contemporary management of pericardial effusion: practical aspects for clinical practice. Postgrad Med.

[8] Tse G, Ali A, Alpendurada F, Prasad S, Raphael CE, Vassiliou V (2015). Tuberculous constrictive pericarditis. Res Cardiovasc Med.

[9] Cunningham-Rundles C, Bodian C (1999). Common variable immunodeficiency: clinical and immunological features of 248 patients. Clin Immunol.

[10] Bonilla FA, Barlan I, Chapel H (2016). International Consensus Document (ICON): Common Variable Immunodeficiency disorders. J Allergy Clin Immunol Pract.

[11] Seidel MG, Kindle G, Gathmann B (2019). The European Society for Immunodeficiencies (ESID) Registry Working definitions for the clinical diagnosis of inborn errors of immunity. J Allergy Clin Immunol Pract.

[12] Hammarström L, Vorechovsky I, Webster D (2000). Selective IgA deficiency (SIgAD) and common variable immunodeficiency (CVID). Clin Exp Immunol.

[13] Gathmann B, Mahlaoui N (2014). Clinical picture and treatment of 2212 patients with common variable immunodeficiency. J Allergy Clin Immunol.

[14] Sharma NK, Waymack JR. Acute Cardiac Tamponade. StatPearls. StatPearls Publishing: Treasure Island (FL);; 2022. 30521227

[15] Stashko E, Meer JM. Cardiac Tamponade. StatPearls. StatPearls Publishing: Treasure Island (FL);; 2022.

[16] Pérez-Casares A, Cesar S, Brunet-Garcia L, Sanchez-de-Toledo J (2017). Echocardiographic evaluation of Pericardial Effusion and Cardiac Tamponade. Front Pediatr.

[17] Sharma PR, Jain S, Bamezai RNK, Tiwari PK (2010). Utility of serum LDH isoforms in the assessment of mycobacterium tuberculosis induced pathology in TB patients of Sahariya tribe. Indian J Clin Biochem.

[18] Baul SK, Hossain SMR, Parvin D, Hadiuzzaman M, Islam MS, Fatema K (2017). Evaluation of LDH and Gamma Interferon as biochemical markers for diagnosis of Pulmonary and Extra-pulmonary Tuberculosis. Bangladesh J Med Biochem.

[19] Monach PA (2014). Biomarkers in vasculitis. Curr Opin Rheumatol.

[20] López-López JP, Posada-Martínez EL, Saldarriaga C (2021). Tuberculosis and the heart. J Am Heart Assoc.

[21] Reuter H, Burgess LJ, Louw VJ, Doubell AF (2007). The management of tuberculous pericardial effusion: experience in 233 consecutive patients. Cardiovasc J S Afr.

