# Supplementary material for: Shorter antitubercular therapy for extrapulmonary tuberculosis - a case report
Source: BMC Infect Dis. 2024 Jan 15;24:86. doi: 10.1186/s12879-023-08941-2 (PMC10788989; doi:10.1186/s12879-023-08941-2)
Supplement: Supplementary file 1 — Supplementary Material 1 [file 12879_2023_8941_MOESM1_ESM.docx]

**DATA**

Laboratory values on the day of admission and trending

| Parameters | Day-1 | Day-2 | Day-3 | Units |
| --- | --- | --- | --- | --- |
| 1. CBC | | | | |
| Hemoglobin | 8.0 | 6.1 | 8.4 | gm/dL |
| RBC count | 3.66 | 2.86 | 3.43 | millions/microlitere |
| WBC count | 16,200 | 12,800 | 5,600 | per microlitre |
| Platelet count | 232,000 | 322,000 | 148,000 | per microlitre |
| Hematocrit | 25.8 | 19.5 | 26.3 | % |
| Mean corpuscular volume (MCV) | 70 | 68 | 77 | femtolitre |
| Mean corpuscular hemoglobin concentration (MCHC) | 21.8 | 31.6 | 31.9 | gm/dL |
| Absolute neutrophil count (ANC) | 12,640 | 10.87 | 3,770 |  |
| Erythrocyte sedimentation rate (ESR) |  | 100 |  | mm/hour |
| 1. Iron studies | | | | |
| Iron |  | 45.12 |  | micrograms/dL |
| Total iron binding capacity (TIBC) |  | 242.8 |  | micrograms/dL |
| Transferrin saturation |  | 19 |  | % |
| Ferritin |  | 362.8 |  | micrograms/litre |
| 1. Basic metabolic panel (BMP) | | | | |
| Blood urea nitrogen (BUN) | 17 | 31 | 52 | mg/dL |
| Creatinine | 2.74 | 4.38 | 1.94 | mg/dL |
| Urine protein |  |  | 25.2 | mg/dL |
| Urine creatinine |  |  | 78.47 | mg/dL |
| Urine protein:creatinine ratio (UPCR) |  |  | 0.32 |  |
| Serum uric acid |  | 8.5 |  | mg/dL |
| Sodium (Na+) | 132 | 135 | 132 | mmol/L |
| Potassium (K+) | 4.2 | 4.4 | 3.8 | mmol/L |
| Calcium (Ca+2) |  | 6.8 |  | mg/dL |
| Magnesium (Mg+2) |  | 1.5 |  | mg/dL |
| Phosphorus |  | 6.1 |  | mg/dL |
| 1. Liver function tests (LFTs) | | | | |
| AST/SGOT | 44 | 185 | 59 | U/L |
| ALT/SGPT | 13 | 473 | 255 | U/L |
| GGT | 73 | 53 | 77 | U/L |
| ALP | 93 | 73 | 66 | U/L |
| Total bilirubin | 1.24 | 0.36 | 0.58 | gm/dL |
| Direct bilirubin | 0.95 | 0.28 | 0.30 | gm/dL |
| Indirect bilirubin | 0.29 | 0.08 | 0.28 | gm/dL |
| Serum protein | 3.9 | 4.2 | 4.2 | gm/dL |
| Serum albumin | 2.9 | 2.8 | 2.9 | gm/dL |
| Albumin:globulin ratio | 2.9 | 2.0 | 2.2 |  |
| 1. Miscellaneous | | | | |
| Serum troponin-T levels | 22.37 |  |  | ng/mL |
